# Automatic Segmentation of Periodontal Tissue Ultrasound Images with Artificial Intelligence: A Novel Method for Improving Dataset Quality

**DOI:** 10.3390/s22197101

**Published:** 2022-09-20

**Authors:** Radu Chifor, Mircea Hotoleanu, Tiberiu Marita, Tudor Arsenescu, Mihai Adrian Socaciu, Iulia Clara Badea, Ioana Chifor

**Affiliations:** 1 Department of Preventive Dentistry, University of Medicine and Pharmacy Iuliu Hatieganu, 400083 Cluj-Napoca, Romania; 2 Chifor Research SRL, 400068 Cluj-Napoca, Romania; 3 Romanian Institute of Science and Technology, 400022 Cluj-Napoca, Romania; 4 Computer Science Department, Technical University of Cluj-Napoca, 400114 Cluj-Napoca, Romania; 5 Department of Radiology and Imaging, University of Medicine and Pharmacy “Iuliu Hatieganu”, 400162 Cluj-Napoca, Romania

**Keywords:** periodontal tissue, automatic segmentation, artificial intelligence, dataset quality, 3D ultrasound reconstructions

## Abstract

This research aimed to evaluate Mask R-CNN and U-Net convolutional neural network models for pixel-level classification in order to perform the automatic segmentation of bi-dimensional images of US dental arches, identifying anatomical elements required for periodontal diagnosis. A secondary aim was to evaluate the efficiency of a correction method of the ground truth masks segmented by an operator, for improving the quality of the datasets used for training the neural network models, by 3D ultrasound reconstructions of the examined periodontal tissue. Methods: Ultrasound periodontal investigations were performed for 52 teeth of 11 patients using a 3D ultrasound scanner prototype. The original ultrasound images were segmented by a low experienced operator using region growing-based segmentation algorithms. Three-dimensional ultrasound reconstructions were used for the quality check and correction of the segmentation. Mask R-CNN and U-NET were trained and used for prediction of periodontal tissue’s elements identification. Results: The average Intersection over Union ranged between 10% for the periodontal pocket and 75.6% for gingiva. Even though the original dataset contained 3417 images from 11 patients, and the corrected dataset only 2135 images from 5 patients, the prediction’s accuracy is significantly better for the models trained with the corrected dataset. Conclusions: The proposed quality check and correction method by evaluating in the 3D space the operator’s ground truth segmentation had a positive impact on the quality of the datasets demonstrated through higher IoU after retraining the models using the corrected dataset.

## 1. Introduction

Periodontal disease is an infection resulting in inflammatory conditions that affect the supporting structures of the teeth (the gingiva, bone, and periodontal ligament), which could lead to tooth loss and contribute to systemic inflammation [1]. The periodontal diseases are highly prevalent and can affect up to 90% of the worldwide population [2]. The diagnosis process is complex, time consuming, and based on manual periodontal probing, which is operator dependent [3,4,5,6] and on radiological investigations, which are invasive and provide a lack of soft tissue contrast [7,8,9,10]. Other investigations that may add value to the diagnosis process are periodontal biomarker analysis [11] and microbiological analysis [12,13,14]. Due to the evolution of technology, periodontal tissue imaging has started to play a key role in periodontal disease diagnosis. Previous studies showed efficiency for non-invasive periodontal imaging investigations using optical coherence tomography [15,16,17] and periodontal ultrasonography [18,19]. The main inconvenience for non-invasive or X-ray imaging technologies is the operator dependence and time-consuming interpretation process. To overcome this drawback, artificial intelligence (AI) may be used for the automatic interpretation of the obtained images. X-ray investigations’ automatic interpretation may be successfully performed for periodontal disease diagnosis using convolutional neural networks (CNNs) [20]. The invasiveness generated by the ionizing radiation [21] and the soft tissue poor visualization [7,21] limit the usage of radiographs for monitoring the disease evolution or treatment results. Ultrasound imaging has a great potential to become a powerful tool for periodontal disease diagnostics [22]. The problem is that the periodontologists and dentists are not used to interpreting ultrasound images of periodontal tissue, representing a true challenge for them [23]. The automatic segmentation based on artificial intelligence helps the operator to speed up the interpretation process, especially for the ones who are not familiar with this type of image interpretation. This will greatly assist dental practitioners to provide better point-of-care to patients [23,24]. 

This research aimed to evaluate Mask R-CNN and U-Net automatic segmentation based on pixel-level classification of bi-dimensional US dental arch images for identifying anatomical elements required for periodontal diagnosis. A secondary aim was to evaluate the efficiency of a correction method of the ground truth data of the masks segmented by an operator, for improved diagnosis or for generating high quality datasets, using 3D ultrasound reconstructions of the examined periodontal tissue.

The main contributions of this paper are the in-house datasets generated by our team, containing thousands of periodontal ultrasound images. Those datasets were used for the training and prediction of U-NET and Mask R-CNN models. Furthermore, a novel method of a quality check and correction of the 2D periodontal ultrasound ground truth image dataset by semi-automatic segmentation and 3D reconstructions was developed.

## 2. Materials and Methods

### 2.1. Data Acquisition and US Image Processing

Fifty-two teeth and marginal periodontium from dental arches of 11 patients, 5 males and 6 females, aged between 20 and 68 years old, were scanned using a 3D ultrasound scanner prototype based on a high frequency standard ultrasound (US) machine (Vinno 6, Suzhou, China, with a 12.8 mm, 10–23 MHz linear transducer, X10-23L, Vinno, China) and a pose reading sensor (an articulated measurement arm Evo 7, RPS Metrology, Sona/Verona, Italy). The prototype was developed by fixing the US transducer at the probe level of the RPS articulated measuring arm and performing spatial and temporal calibration of the two devices [25]. One of the eleven patients had stage 4 grade C generalized periodontitis, two patients had stage 3 grade B generalized periodontitis, two patients had stage 2 grade B, and six were periodontally healthy patients. Natural teeth without carious lesions or reconstructions were included in the study. The scanning procedure was performed by an experienced operator in periodontal ultrasonography, having the transducer in a longitudinal position, aligned with the long axis of the tooth, placed on the patient’s face skin. The buccal surface of the teeth was scanned moving the transducer in a mesial direction starting from the right upper second premolar and going clockwise to the right lower second premolar (hemiarch 1, 2, 3, and 4). The bi-dimensional periodontal ultrasound images were acquired in standard DICOM files at 20 Mhz. After acquisition, the 2D US periodontal images were extracted using an in-house developed python software application and were saved in PNG files. A total of 3417 periodontal US images were selected to form the datasets for training the AI models. The selected images were from dental and interdental spaces. Noisy or low quality periodontal US images were excluded. The selection of the images was performed by an experienced operator in periodontal ultrasonography. The study was carried out under the Iuliu Hatieganu University of Medicine and Pharmacy, Cluj-Napoca, Romania, with ethical approval (197/06.07.2022). Informed consent was obtained from all subjects involved in the study.

**Figure 1:**
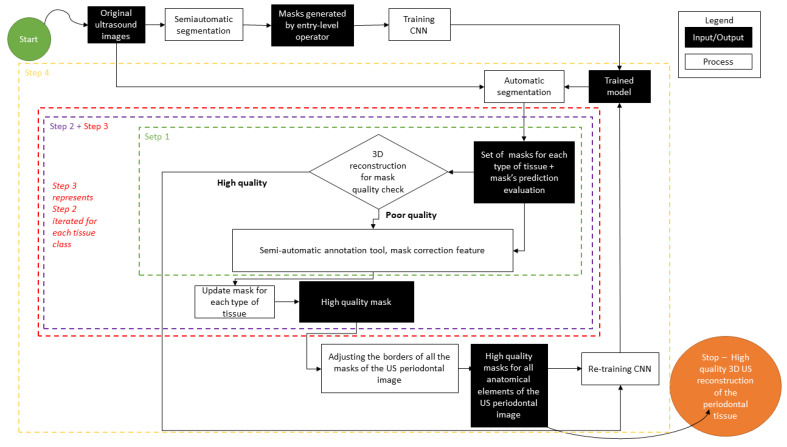
Mask generation process and quality check in the 3D space.

The original US images were processed following the steps described in Figure 1. A semi-automatic segmentation was performed by a young dentist, with low experience in periodontal US image processing (after 2 h of training). Five anatomical elements (tooth crown, tooth root, cortical bone, gingiva, periodontal pocket) were searched for and identified if present. Every anatomical element has been masked using an in-house developed semi-automatic software annotation tool.

**Figure 2:**
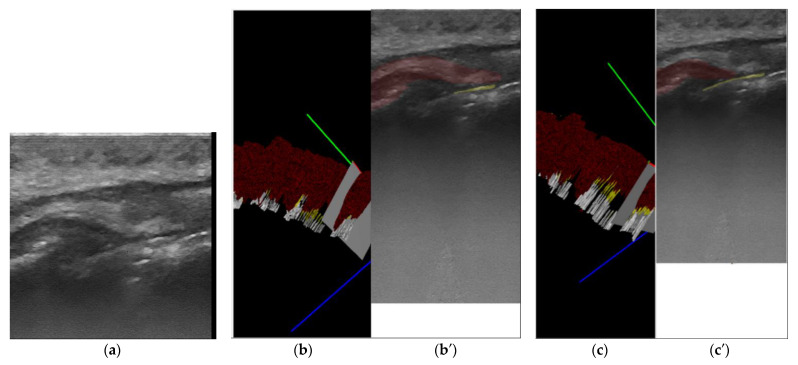
(**a**) 1.4, first upper right premolar, longitudinal section of the marginal periodontal tissue, buccal surface original US image, gray scale; (**b**) 3D visualization: red, green, blue lines—cross-sectioning plane direction on the 3 axes; (**b′**). first semi-automatic segmentation: white—tooth crown, yellow tooth root, dark red—gingiva, light red—cortical bone; (**c**) 3D visualization using corrected masks; (**c′**) segmentation after correction: white—tooth crown, yellow tooth root, dark red—gingiva, light red—cortical bone.

**Figure 3:**
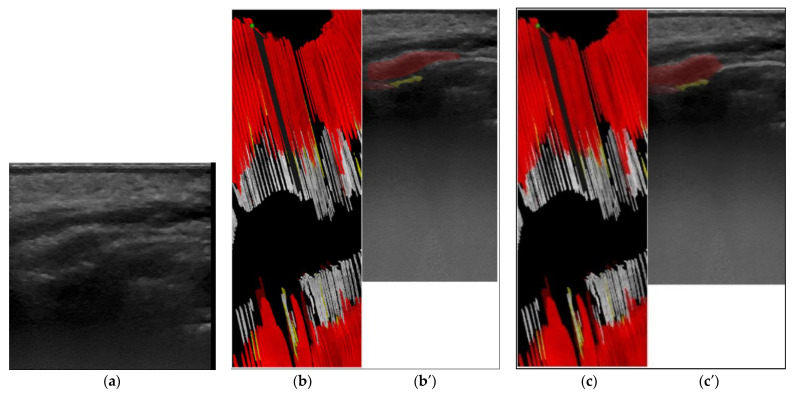
(**a**) 2.1, central incisor, longitudinal section of the marginal periodontal tissue, buccal surface original US image, gray scale; (**b**) 3D visualization: red—cross-sectioning plane direction orientation on *X* axis; (**b′**). first semi-automatic segmentation: white—tooth crown, yellow–tooth root, dark red—gingiva, light red—cortical bone; (**c**) 3D visualization using corrected masks; (**c′**) segmentation after correction: white—tooth crown, yellow tooth root, dark red—gingiva, light red—cortical bone.

The second step was to generate a 3D reconstruction of the scanned area using the original 2D US images, masked with the result from the semi-automatic annotation, and the 3D calculated position of the original images. The quality check for the masks (Figure 1) was performed in the 3D space by the same operator with low experience in periodontal ultrasonography. The operator can navigate through the 3D ultrasound reconstruction frame by frame identifying the errors. The masks that did not fit the 3D object were corrected in the 2D images using the semi-automatic segmentation tool. The last step was retraining the artificial intelligence model using the modified dataset after corrections and evaluation of the result once again in the 3D space (Figure 2 and Figure 3). Unregulated areas in tooth surfaces, tooth root, or crown can be easily seen after 3D reconstruction, pointing out artifacts or errors in masking the periodontal anatomical elements.

**Figure 4:**
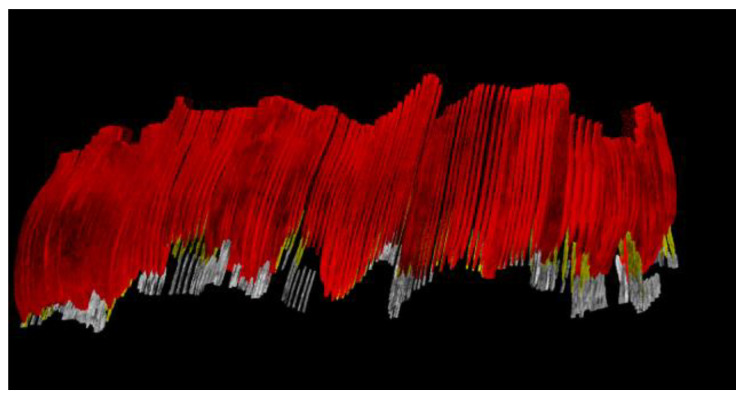
Three-dimensional reconstruction of maxillary arch, marginal periodontal tissue, buccal surface of the gingiva—red, crown surface—white, root surface—yellow. One can easily see areas where the gingival contour and margin is unregulated, showing artifacts or errors in masking the periodontal anatomical elements.

Artifacts or segmentation errors are clearer in the 3D space (Figure 4), showed by unregulated areas of the reconstructed tissues. If the masks are correct, having low artifacts on the surfaces of the gingival tissue, root, crown, and bone should appear continuous.

The components and features of the in-house developed software, some of them being detailed in previous studies [25,26], are: the semi-automatic annotation tool for generating the initial dataset from original US images and the automatic image segmentation tool based on pixel-level classification using Mask R-CNN and U-Net models; 3D ultrasound reconstruction and visualization software of bi-dimensional masked or unmasked US images; cross-section features of the 3D ultrasound reconstruction of the scanned area; and navigation through the 3D ultrasound reconstruction frame by frame for evaluation and mask correction. 

From all the 3417 selected images, 2135 were included in the datasets for training the AI models before and after correction. Those images were acquired from 32 teeth and marginal periodontium from dental arches of 5 patients. One patient had stage 4 grade C generalized periodontitis, one patient had stage 3 grade B generalized periodontitis, one patient had stage 2 grade B, and two were periodontally healthy patients.

The datasets:

- Dataset 12—1370 files (MR-CNN, U-NET, train files: 1051, validation files: 131, test files: 132).
- Dataset 12 c—corrected after a quality check in the 3D space.
- Dataset 125—2135 files (MR-CNN, U-NET, train files: 1708, validation files: 213, test files: 214).
- Dataset 125 c—corrected after a quality check in the 3D space.
- Dataset 12345—3417 files (MR-CNN, U-NET, train files: 2733, validation files: 341, test files: 343).

### 2.2. The Semi-Automatic Annotation Tool

The region growing algorithm was used to create a semi-automatic segmentation of periodontal tissues in ultrasound images. It was based on the breadth-first search (traversal) algorithm of graphs [27] and uses a queue structure (FIFO list) for optimal implementation. The first implementation attempt was undertaken in C to allow the compiler to compensate for un-optimized looping and conditional branching constructs. 

The steps of the proposed method for semi-automatic annotation of the images based on the region growing algorithm are described below. 

The user must click on some seed points which will be “grown” by iteratively adding neighboring pixels with similar intensity. The algorithm is customized in such a way that already labeled pixels are not considered. The similarity predicate is controlled by a threshold T tunable by the user. 

**Figure 5:**
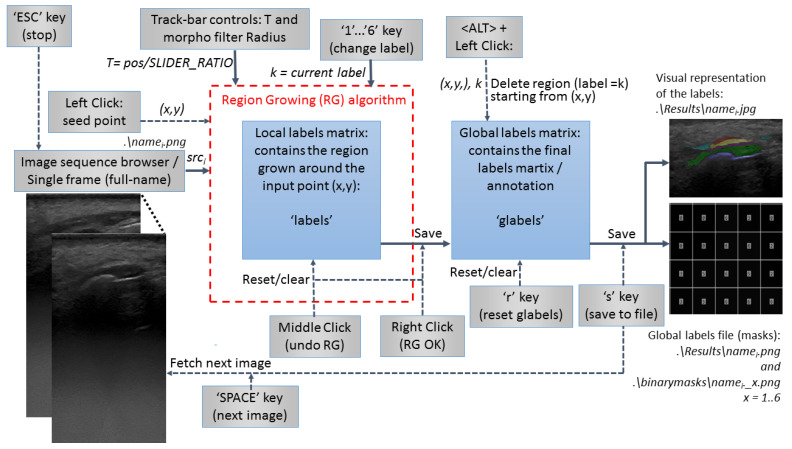
Flow chart of the semi-automatic annotation tool.

The flow chart of the application is presented in Figure 5. It interacts with the user through the keyboard and the mouse as well as through the OpenCV GUI for displaying the input and output images and to control a Track-bar used to set the threshold T for region growing.

The sequence of operations needed to annotate an image is (default label at the application start is 1):

1. Click on a relevant point of the anatomical region corresponding to label/class 1.
2. Adjust the region growing threshold (T) through the slider of the Track-bar. If the operator wants to undo the last local region growing (mouse Right_Button was not clicked): Left_Button_Click on an unlabeled pixel which will be the new seed point or Middle_Button_Click (anywhere on the image).
3. When satisfied with the local segmentation, Right_Button_Click to save it in the global label matrix. After a Right_Button_Click or a Middle_Button_Click, the Track-bar slider has no effect. A region is grown and can be adjusted through the Track-bar only after a new seed is selected by Left_Button_Click (grow_in_progress = true).

The same tool was used for masks’ corrections after a quality check in the 3D space. The functionalities of eraser and pencil draw were also available. 

### 2.3. The Image Segmentation Method Based on Pixel-Level Classification

The acquired periodontal US images were 375 × 735 pixel PNG files. The segmentation should identify 5 anatomical elements: bone, crown, gingiva, periodontal pocket, and root. Each image may contain 0, 1, or more instances of each object (anatomical element). The target was to perform a semantic segmentation.

To generate the datasets for training purposes, a large set of images were semi-automatically segmented by an operator with low experience in periodontal US. For each ultrasound image, a set of up to 5 mask files were created, each of them containing the mask for a specific object—if that object is visible in the ultrasound image.

Using the original US periodontal images and the masks, Mask R-CNN and U-NET deep learning models were trained and tested to automatically identify the objects in the ultrasound images. The results were tested for evaluation of the overlapping between the prediction and operator segmentation, considered the ground truth (IoU—Intersection over Union), segmentation speed, accuracy, sensitivity, and specificity of the predictions.

**Figure 6:**
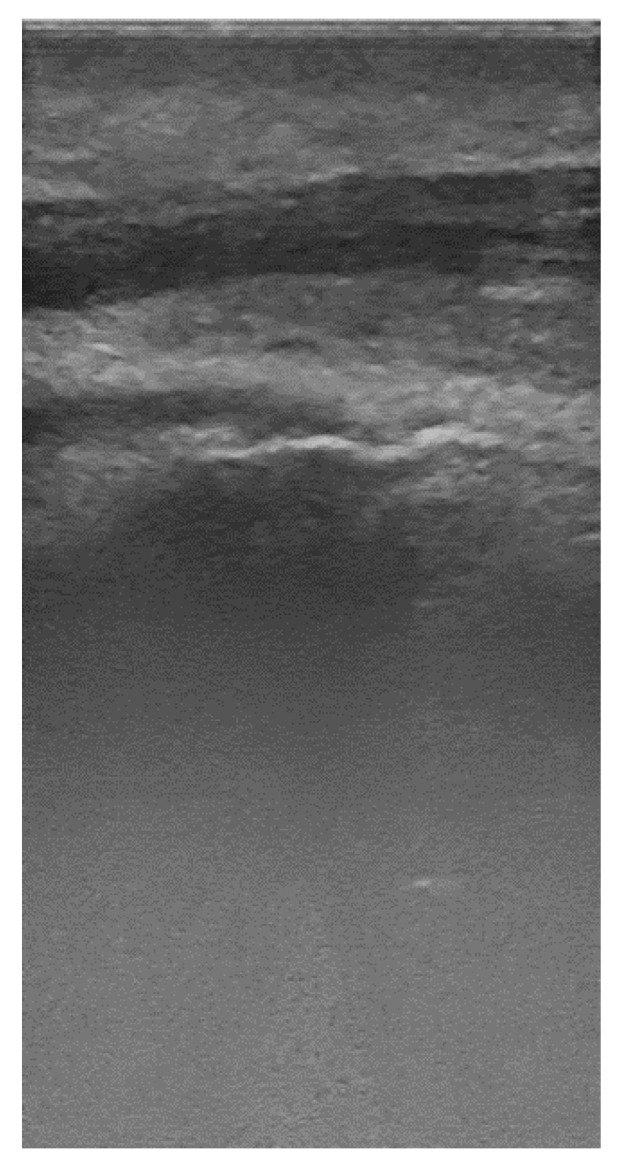
Upper premolar, periodontal ultrasound image, 20 MHz.

A typical mandibular and maxillary ultrasound image is presented in Figure 6. As one can see in Figure 6, the images are characterized by low contrast and high noise. Moreover, browsing through several images, it can be noticed that only the upper half of the image contains the relevant information (all the anatomical elements are in the upper part of the image).

The images may contain up to five elements: bone, crown, gingiva, periodontal pocket, and root.

**Figure 7:**
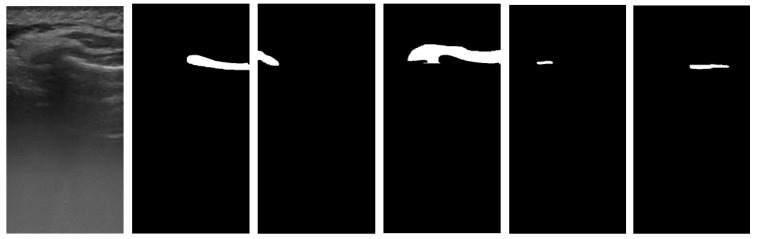
From left to right: original ultrasound image, bone, crown, gingiva, pocket, root.

The annotation consists of binary images of 375 × 735 pixels, with each image being a mask corresponding to one of the elements present in the images (as seen in Figure 7).

#### Deep Learning Models for Image Segmentation

To decide the implementation of a deep learning model in this project, the following requirements had to be met: highly accurate pixel classification with a reasonable speed. 

Most of the deep learning models used for image segmentation are based on convolutional neural networks (CNNs). Some of the biggest software companies have already developed very powerful models for this task which proved excellent performance for general purpose applications, mostly object detection in photography. However, the medical applications involve images that are far less sharp and have lots of noise. For that reason, many other CNN topologies have been demonstrated. 

Mask R-CNN was developed by Facebook, and it is considered today one of the most powerful instance segmentation (semantic segmentation + delimitation of individual objects) models. Taking advantage of Facebooks’ huge computing power, the model was trained using hundreds of thousands of images and in its vanilla form it is able to identify about 100 different objects classes.

- Benefits: It is reported as having one of the highest accuracies and can be used as a backbone for extending the objects to be detected.
- Drawbacks: It is a heavy model (244 MB), is not very fast—a couple of seconds for detection, has a long retraining time, and does not ensure that detected objects are not overlapping.

U-NET is a pure CNN deep learning image segmentation structure specially developed for medical imaging.

- Benefits: It is a simple model, easy to implement and to control, very fast both in training and detecting phases, has reasonable accuracy, ensures the detected objects do not overlap, and has faster detection than Mask R-CNN.
- Drawbacks: It is less accurate compared with Mask R-CNN or DeepLab (developed by Google mostly for automotive applications).

After analyzing the benefits and drawbacks, we decided to proceed with the implementation of the two models: Mask R-CNN and U-NET. 

All programs described below are written in Python 3.7, with TensorFlow 1.2.1 and Keras 2.1. The programs were run on NVIDIA GPUs with CUDA 10.2.

- Implementation of the application set using Mask R-CNN

Mask R-CNN deep learning model is an instance segmentation model that provides three outputs: the bounding box of each object instance, its class, and its mask.

It combines CNN and fully connected layers. Specific to this model is the region proposal network, a block that basically is looking for regions where instances may be found. This way, the model can identify a large number of instances faster. 

For training, the input of Mask R-CNN was an RGB image, and the corresponding masks organized in a three-dimensional binary file. This file contained the masks of all instances of all classes identifiable in the image as successive layers having the same width and height as the input RGB image. The model was optimized to work with square images having the dimensions as multiples of 64. 

As a predictor, Mask R-CNN takes one input square RGB image and provides the list of instances identified in the image, their classes, bounding boxes, and pixel masks.

○ Dataset preparation

Datasets were organized as follows:

- Each patient’s data were stored in one set of images.
- Each set included the ultrasound images (.png files) and associated mask files (.png files), one mask file for each anatomical element, detectable in the ultrasound image.

The dataset compatible with the Mask R-CNN model was created using the Python software package performing the following tasks:

- Checking the validity of the files, correcting when possible or eliminating them. The corrections included: file naming errors, file format discrepancies.
- Cropping the images and the mask to the size compatible with Mask R-CNN. Considering that the ultrasound images and the masks have 375 × 735 pixels and that the lower half part of the images do not contain useful information, the images and masks were cropped in a vertical direction and extended in a horizontal direction to a dimension of 384 × 384 pixels.
- The five binary masks were saved in a 384 × 384 × 5 binary file with the predefined order of elements: bone, crown, gingiva, pocket, root. If a certain element was not present in the image, the corresponding layer in the binary file was left empty (zeros).

For the development phase, each dataset was split in three: training dataset, validation dataset, and test dataset.

○ Model training

To train the Mask R-CNN model, a Python program that implements the model using the Resnet60 backbone was created. 

A specific class to load our dataset with our predefined list of five elements was developed. The program can load all the files from a specific location or specific files contained in a text file. The latter option is useful for testing various model configurations using the same training dataset.

The program allowed us to tune various model parameters in order to optimize its performance, such as batch size, number of epochs, number of regions, number of trainable layers, learning rate, etc. 

The program also allowed the use of multiple GPUs to speed up the training and the predictions. 

The training default parameters were loaded from a model trained with the MS COCO dataset. That ensured a faster training. The trained models were saved in .h5 files.

○ Predictions

To generate the predictions, a Python program was developed that loads the saved models then allows the user to input RGB images prepared to fit the required 384 x 384 pixel size. 

The results were available in binary files of format 384 × 384 × (no. of instances) and the list of classes corresponding to all predicted instances. Because the interest was not in instance segmentation, but rather in semantic segmentation, all the instances of a certain class were consolidated into a single mask and that mask was saved on this for later use.

○ Tests

A separate Python program was developed to measure the prediction accuracy. 

The prediction accuracy was measured by two parameters: precision of instance prediction and pixel precision IoU (Intersection over Union). The instance precision measured the number of true positive, true negative, false positive, and false predicted negative instances as compared with the operator’s semi-automatic annotation. IoU was measured for true positive instances as the ratio between the number of overlapping pixels and the union of the pixels from prediction and operator’s semi-automatic annotation.

- Implementation of the application set using U-NET

U-NET is a fully convolutional neural network design to apply especially for semantic segmentation of medical images. It consists of two paths: one contracting and one expanding. Characteristic to U-NET are also the skip paths connecting the contracting and expanding paths.

○ Dataset preparation

The U-NET model accepts as a dataset the same files as Mask R-CNN, and for that reason we did not create a separate dataset for it.

○ Model training

U-NET has been originally designed to handle training with a small size dataset. Because of that, to minimize the risk of overfitting, the standard implementation has a reduced convolutional layer size and includes drop layers. In our case, we had a rather large dataset (a couple of thousand images). That allowed us to modify the model by increasing the size of the convolutional layers and by reducing the drop layers. 

So, our program allowed us to tune the layers’ size and set the number of drop layers. In addition, the program allowed us to set the batch size and the learning rate. The trained model was saved as a h5 file.

○ Predictions

To generate the predictions, a Python program was developed. The program loads the saved models and then allows the user to input RGB images prepared to fit the required 384 × 384 pixel size. 

The results were available in binary files of format 384 × 384 × 5, with one layer for each detected element.

○ Tests

As for the Mask R-CNN model, a separate Python program was developed to measure the prediction accuracy for U-NET. The prediction accuracy was measured by two parameters: precision of instance prediction and pixel precision IoU (Intersection over Union). The instance precision measured the number of true positive, true negative, false positive, and false predicted negative instances as compared with the manual annotation. IoU was measured for true positive instances as the ratio between the number of overlapping pixels and the union of the pixels from prediction and manual annotation.

## 3. Results

### 3.1. Optimization of the Mask R-CNN Model

Considering the resource constraints, the focus was on optimizing the following parameters: number of trainable layers, number of epochs, and number of regions.

- Optimizing the number of trainable layers

Because the default model parameters were trained using the MS COCO dataset, the default parameters were not able to detect the elements in the ultrasound images. No other default parameters were available for our dataset. As a result, the transfer learning technique was used and retrained the whole model (not only the output layers) starting from the MS COCO parameters.

- Optimizing the number of epochs

**Figure 8:**
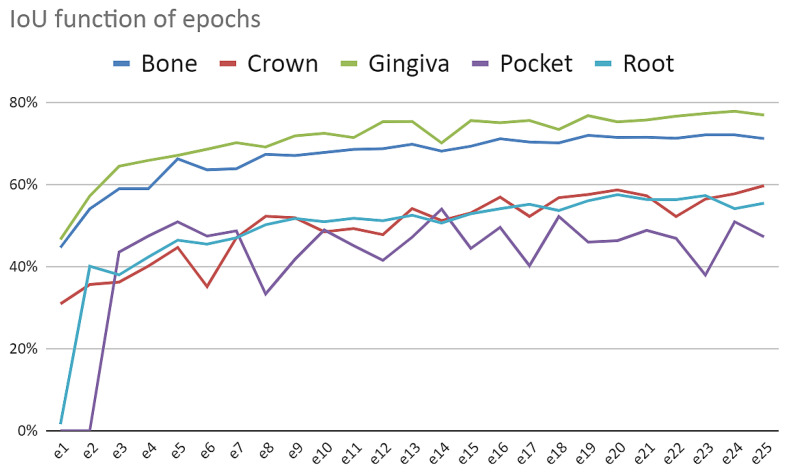
Mask R-CNN accuracy (IoU) as a function of number of epochs.

As can be seen in Figure 8, the number of IoU is flattened after about 20 epochs. Furthermore, there is no sign of overfitting. The behavior was the same for all tests performed. For that reason, it was concluded that the optimum number of epochs is 20.

- Optimizing the number of zones

**Figure 9:**
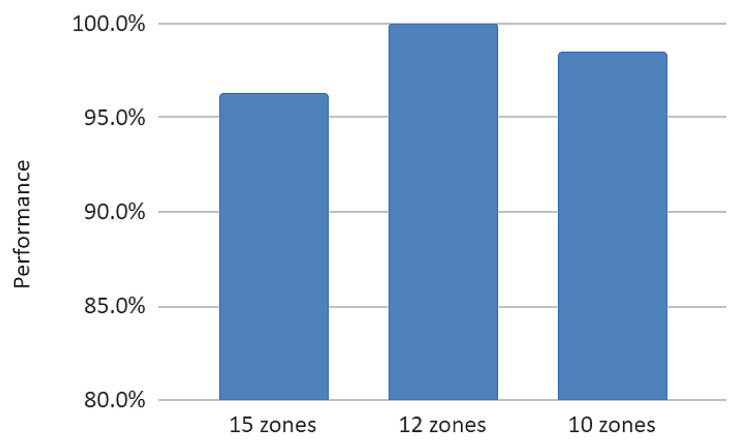
Model performance as a function of number of zones.

According to the Mask R-CNN developers, the number of zones should be set to values about 3 times the number of expected elements in the images. In our case, the maximum number of elements are five, but in many cases some of the elements are missing. To determine the optimum number of zones, the model was trained with the same dataset for three different settings: 15 zones, 12 zones, and 10 zones. The performance of the trained models was measured by calculating the average IoU across all elements and normalizing the results to the best value (Figure 9). As one can see, the optimum number of zones is 12.

### 3.2. Optimization of the U-NET Model 

The U-NET model was optimized by tuning the layers’ size and the number of drop layers.

- Optimizing the layers’ size

**Figure 10:**
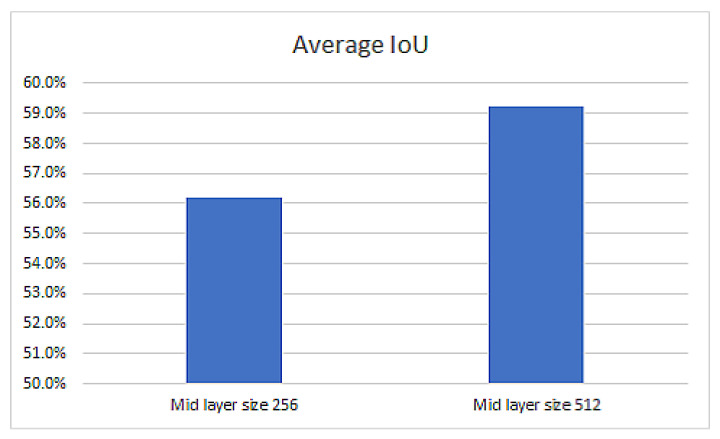
U-NET performance as a function of model size.

Two U-NET models were trained, with one having the middle layer of size 256 (1,940,885 parameters) and the other one with size 512 (7,759,653 parameters). The results (Figure 10) shows that a higher dimension produces more accurate results.

- Optimizing the number of drop layers

**Figure 11:**
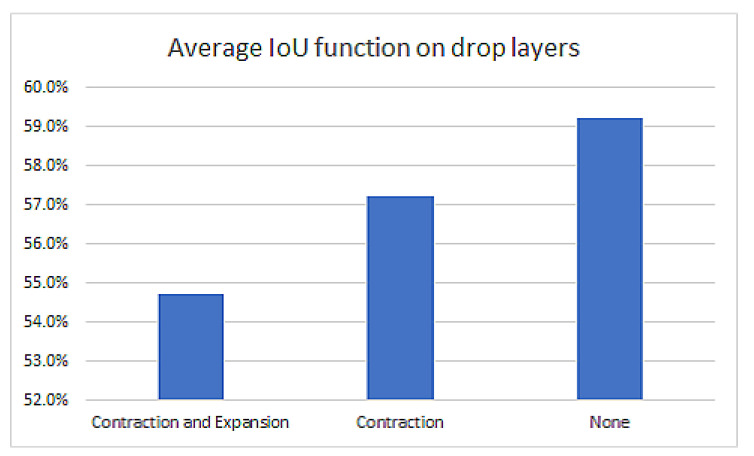
U-NET performance as a function of employed drop layers.

The drop layers are commonly used in U-NET models trained with a small dataset to avoid overfitting. The effect of the drop layers was tested for our dataset on the large model (middle layer size 512) in three different situations: drop layers after all CN layers, drop layers only in the contraction branch, and without any drop layers. The results are presented in Figure 11. We notice that the best performance is obtained on the model without drop layers.

### 3.3. Summary of Model’s Performance

- Overall comparison on Mask R-CNN vs. U-NET performance

**Figure 12:**
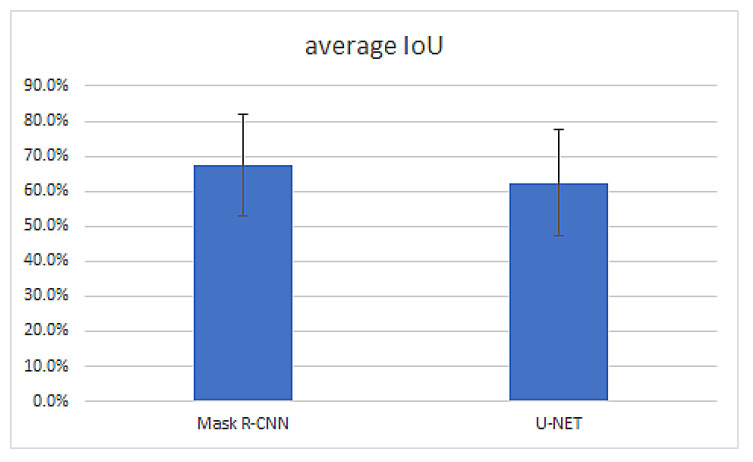
Weighted average IoU for Mask R-CNN and U-NET.

Mask R-CNN performance measured as IoU is better than U-NET Performance. Figure 12 shows the weighted average IoU of the best Mask R-CNN and U-NET models.

- The effect of the dataset accuracy

**Figure 13:**
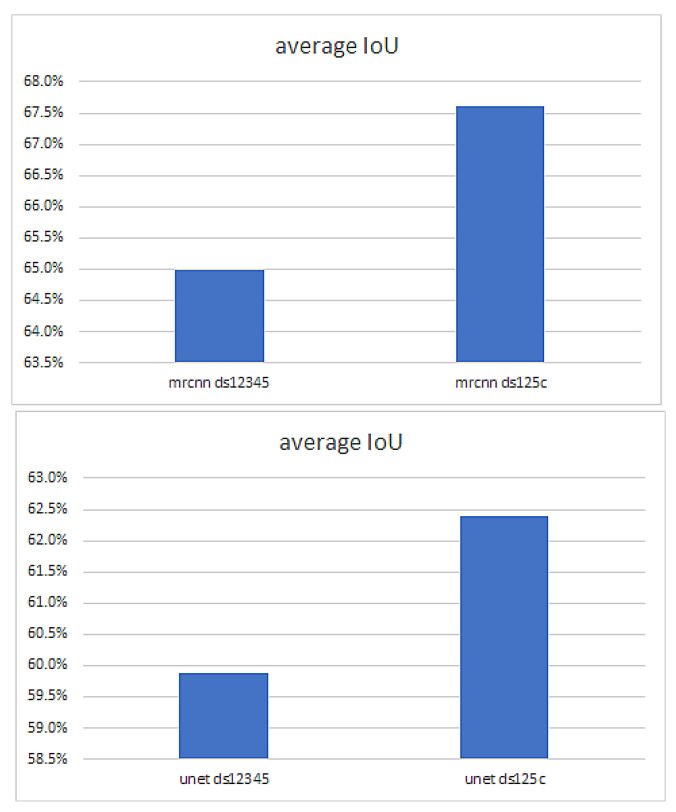
The effect of the annotation accuracy tested on both Mask R-CNN (**above**) and U-NET (**below**) for an uncorrected dataset (**left**) vs. a corrected dataset (**right**).

**Figure 14:**
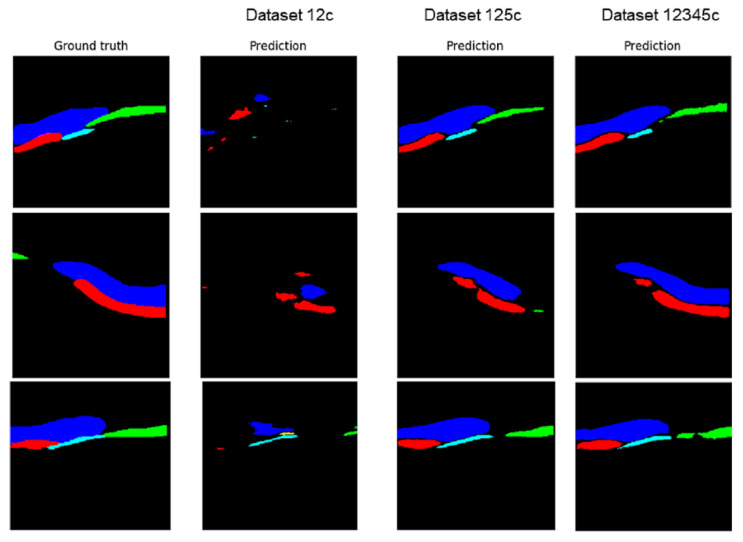
Prediction using the U-NET trained model with different datasets (DS12C, DS125C, DS12345C). Ground truth—high quality segmentation, undertaken by the operator after analyzing the 3D reconstructions (corrected dataset). Dark blue—gingiva, green—tooth crown, red—bone, light blue—tooth root.

**Figure 15:**
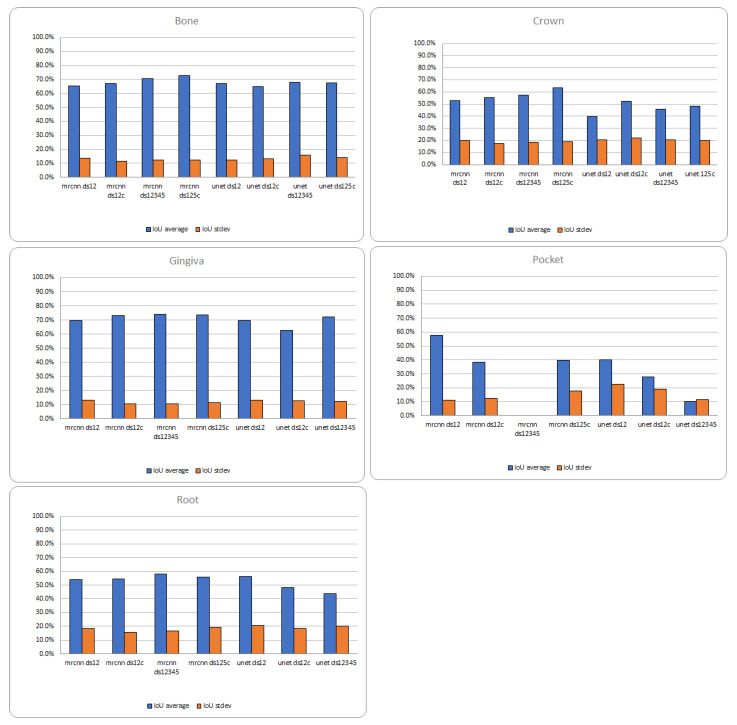
Mask R CNN and U-NET trained with different datasets: accuracy prediction evaluation using IoU average and standard deviation.

One can expect that the quality of the annotations will affect the performance of the predictions. The largest dataset included annotated images, made by a low-level experienced operator, from the scans of 11 patients (DS12345). The annotations of the scans from the patients 1–5 (DS125) have been reviewed and corrected, by the same operator, according to the quality check method using ultrasound reconstructions in the 3D space, described in Figure 1. The masks that did not fit the 3D object were corrected (DS125c). Both Mask R-CNN and U-NET were trained in both situations (before and after corrections). The results are presented in Figure 13, Figure 14 and Figure 15. Even though the original dataset (DS12345) contained 3417 images from 11 patients, and the corrected dataset (DS 125c) only 2135 images from 5 patients, the prediction accuracy is significantly better for the models trained with the corrected dataset (Figure 14 and Figure 15).

Qualitative analysis of the predictions performed by automatic segmentations using the U-NET model trained with different corrected datasets is represented in Figure 14. One can easily see that the size of the dataset used for training directly influence the quality of the segmentation but also the quality of the datasets.

Quantitative analysis, through IoU evaluation, of the predictions performed by automatic segmentations using the Mask R-CNN and U-NET models trained with different corrected and not corrected datasets are represented in Figure 15. The best results are obtained for the largest corrected dataset, with the IoU values being directly proportional to the size of the predicted mask.

For the crown, root, and pocket, there was a possibility or not for the tissue to be present in the original image and the results showed that the quality and the size of the datasets influence directly the quality of the automatic segmentation (Table 1). The specificity for gingival tissue and bone could not be calculated in most of the situations (division by 0 error marked in the table by symbol “-“) because these types of tissues were present in all of the images and the algorithm predicted the presence of the object as 100% (Table 1).

- Other considerations

○ Prediction times for both models are strongly dependent on the computer used for running the model. For Mask R-CNN, the prediction may range from several seconds if run on a CPU to lower than 0.5s if run on a GPU.

Being much lighter, the U-NET prediction is anyway very fast, reaching several predictions per second. 

○ The dataset is very unbalanced, both in terms of presence of elements in the images and the average mask size for elements. The plots below have been calculated using the best Mask R-CNN model trained with the available corrected dataset. 

**Figure 16:**
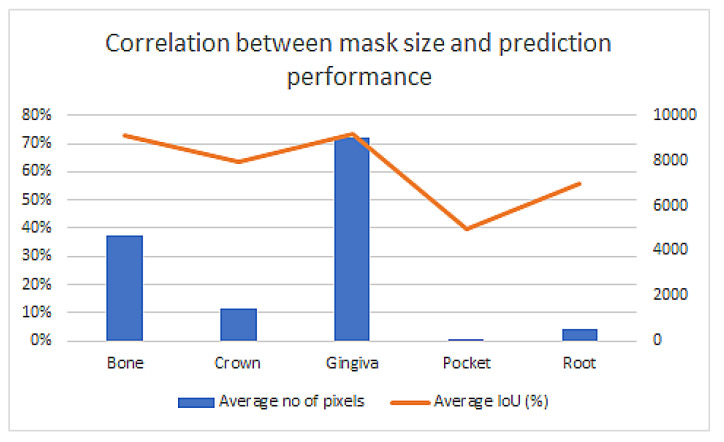
The correlation between the element size (in pixels) and the prediction accuracy.

Figure 16 shows that the prediction accuracy strongly correlates with the average size of the element in the dataset.

**Figure 17:**
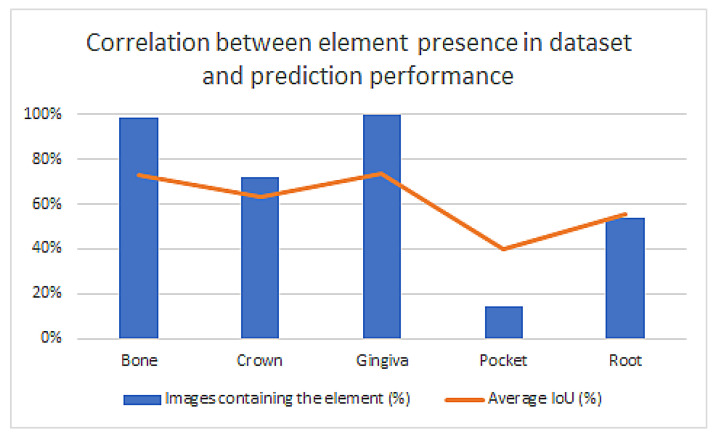
The correlation between the number of element presence in dataset and the prediction accuracy.

Figure 17 shows that the prediction accuracy strongly correlates with the number of elements present in the dataset.

## 4. Discussion

As far as we know, this is the first time when all the necessary anatomical elements for periodontal diagnosis were automatically identified in human ultrasound images. Previous studies focused on a single [23] or several elements [24], but not all. This was the biggest challenge because manual or semi-automatic segmentation to prepare the datasets for training is time consuming and needs a lot of experience from the operator. Moreover, the periodontal US images may contain up to five elements: bone, crown, gingiva, periodontal pocket, and root. There are large variations of characteristics in these elements, especially in terms of their size and occurrence. The periodontal pocket was present only in about 10% of the acquired images in this study, because almost half of the images were from inter-dental spaces where neither the crown, the root, nor the periodontal pocket were present. Another reason is that if the gingival sulcus is very thin, without having sulcular secretion it may not be visible in the original ultrasound image. 

At extremes, in terms of size, gingiva can be tens of times larger than a pocket. Furthermore, gingiva was present in all images, while the pocket was present only in only 10% of the images. These elements make the learning process very difficult. 

To evaluate the classification of different objects in the AI automated segmented images accurately, specificity and sensitivity are usually calculated. For our study, these parameters were not a relevant measure to assess improvements in training and prediction because there are certain types of tissue, such as bone and gingiva, that are always present in the segmented images. Because of this, the trained models identify almost every time correctly, if present or not, the bone and gingiva image segments. Due to that, there were small variations of accuracy, specificity, and sensitivity between different datasets, even if the datasets were small and with poor quality of the ground truth segmentation compared with large datasets and a better quality of operator’s segmentation.

Writz et al.’s findings for U-NET automatic segmentation of teeth in panoramic X-rays [28] in terms of accuracy, specificity, and precision were 0.818, 0.799, and 0.790, respectively. In our study, the results were for the crown automatic segmentation, using U-NET, and were 0.938 for accuracy, 0.922 for specificity, and 0.975 for precision for the ds125C dataset, which was the largest corrected and high quality dataset. For the root automatic segmentation, the accuracy was 0.930, specificity was 0.906, and a precision of 0.951. We consider it a relevant comparison for those two types of tissue because the gingiva and the bone were always present, and the pocket had a very reduced size and was very rare in the images. 

More relevant for our research measurement of improvement in the training and pre-diction of the models would be to use a parameter that gives an indication of size, shape, and position of the segmented tissues compared to the ground truth segmentation. There-fore, we chose IoU (Intersection over Union) for this purpose, because it measures the amount of overlap between the prediction and the ground truth.

The average IoU in our study was a little lower, ranging between 10% for the periodontal pocket and 75.6% for gingiva, comparing it with results obtained by automatic segmentation of teeth contours from dental radiographs using different AI models. In Majanga et al.’s study [29], depending on the AI model, the average IoU ranged between 31.8% and 89%. Jang et al. reported for Mask R-CNN, tooth segmentation in X-ray CBCT 2D images with an average IoU of 92.17% ± 5.5% [30]. The ultrasound periodontal images were far less sharp and had more noise than the X-rays, and in our study were identified five anatomical elements and in theirs only one, which can also impact negatively the IoU. Moreover, the size of our masks was much smaller than the size of a tooth contour, especially for the periodontal pocket. 

We consider that the objectives of this study have been achieved, obtaining through the IoU calculation a quantitative analysis of Mask R-CNN and U-NET automatic seg-mentation of periodontal tissue ultrasound images. The secondary objective was also achieved: using the quality check method in the 3D space of the generated masks, the low experienced operator had the possibility of improving the obtained results without consulting an expert.

The limitations of the study:

While the element size cannot be changed, the strong imbalance due to the number of elements present in the dataset can be reduced by augmentation techniques. A future study may be based on optimized datasets using augmentation techniques. The size of the datasets used for training AI models is very important, having a direct impact on the results of the automatic segmentation. The datasets generated in this study were quite large, but even larger datasets in the future will improve significantly the output quality of the automatic segmentation.

## 5. Conclusions

The proposed quality check and correction method by evaluation in the 3D space of the operator’s ground-truth segmentation had a positive impact on the quality of the datasets. As a result, an improved output of Mask R-CNN and U-NET convolutional neural network models for automatic pixel-level segmentation was obtained. A method like this may help a less experienced operator to generate higher quality datasets in the future. Mask R-CNN had overall better results in automatic segmentation of periodontal tissue in ultrasound images, compared with U-NET, demonstrated by a higher average IoU. The best result in term of overlap between the prediction and ground truth was 75.6% for gingival tissue, which still is not sufficient for a reliable fully automated diagnosis. To achieve a higher quality of the results for automatic segmentation, further studies are necessary using larger datasets for training AI models for segmentation in both the 2D and 3D space. 

## Figures and Tables

**Table 1:** Accuracy, specificity, and sensitivity for the corrected and uncorrected datasets for Mask R-CNN and U-NET predictions.

	MR-CNN	MR-CNN	MR-CNN	MR-CNN	U-NET	U-NET	U-NET	U-NET
	ds12	ds12c	ds12345	ds125c	ds12	ds12c	ds12345	ds125c
*Bone*								
Accuracy	1.000	1.000	0.988	0.981	1.000	0.992	0.994	0.995
Specificity	-	-	0.833	0.000	-	-	0.667	0.667
Sensitivity (Recall)	1.000	1.000	0.991	0.995	1.000	0.992	1.000	1.000
Precision	1.000	1.000	0.997	0.986	1.000	1.000	0.994	0.995
*Crown*								
Accuracy	0.890	0.820	0.858	0.915	0.848	0.921	0.885	0.938
Specificity	0.400	0.462	0.574	0.703	0.700	0.846	0.896	0.922
Sensitivity (Recall)	0.968	0.946	0.969	0.985	0.870	0.944	0.882	0.943
Precision	0.910	0.833	0.853	0.910	0.950	0.952	0.960	0.975
*Gingiva*								
Accuracy	1.000	1.000	0.988	0.995	1.000	0.992	0.994	0.986
Specificity	-	-	0.000	0.000	-	-	0.333	0.000
Sensitivity (Recall)	1.000	1.000	0.997	1.000	1.000	0.992	1.000	0.991
Precision	1.000	1.000	0.991	0.995	1.000	1.000	0.994	0.995
*Pocket*								
Accuracy	0.965	0.936	0.938	0.941	0.969	0.963	0.947	0.959
Specificity	0.976	0.895	0.902	0.972	0.976	0.965	0.938	1.000
Sensitivity (Recall)	0.955	0.975	0.974	0.914	0.963	0.961	0.957	0.925
Precision	0.977	0.907	0.910	0.974	0.977	0.969	0.942	1.000
*Root*								
Accuracy	0.849	0.903	0.881	0.912	0.894	0.910	0.907	0.939
Specificity	0.617	0.786	0.701	0.783	0.702	0.810	0.821	0.906
Sensitivity (Recall)	0.941	0.943	0.960	0.984	0.967	0.944	0.943	0.956
Precision	0.862	0.928	0.879	0.891	0.895	0.936	0.926	0.951

## Data Availability

The datasets from the current study are available from the authors on reasonable request.
